# Effectiveness of Repellent Plants for Controlling Potato Tuber Moth (*Symmetrischema tangolias*) in the Andean Highlands

**DOI:** 10.3390/insects17010024

**Published:** 2025-12-24

**Authors:** Alex Villanueva, Fernando Escobal, Héctor Cabrera, Héctor Cántaro-Segura, Luis Diaz-Morales, Daniel Matsusaka

**Affiliations:** 1 Estación Experimental Agraria Baños del Inca, Dirección de Supervisión y Monitoreo de las Estaciones Experimentales, Instituto Nacional de Innovación Agraria (INIA), Cajamarca 06004, Peru; 2 Centro Experimental La Molina, Dirección de Recursos Genéticos y Biotecnología Instituto Nacional de Innovación Agraria (INIA), Lima 15024, Peru; hcantarosegura@gmail.com; 3 Dirección de investigación y Desarrollo tecnológico (DIDET), Instituto Nacional de Innovación Agraria (INIA), Av. La Molina 1981, Lima 15200, Peru

**Keywords:** potato tuber moth, *Symmetrischema tangolias*, botanical repellents, *Minthostachys mollis*, *Artemisia absinthium*, seed potato storage, integrated pest management

## Abstract

Postharvest losses from potato tuber moth severely constrain seed quality in Andean smallholder systems. This study evaluated four locally available repellent plants—*Ambrosia peruviana*, *Eucalyptus globulus*, *Artemisia absinthium*, and *Minthostachys mollis*—applied as dried leaves layered within seed bags of INIA 302 ‘Amarilis’ under farmer-like storage at two highland sites in Cajamarca, Peru (Huaytorco, 3350 m; Samaday, 2750 m), over 187 days. Within each site, a Completely Randomized Design with three bag-level replicates per treatment was used, and damage was assessed after 187 days as incidence of attacked tubers, internal damage severity and live larval counts. Endpoint data were analyzed separately by site using Kruskal–Wallis tests followed by Dunn’s post hoc test with Šidák correction (α = 0.05). Across both sites, all botanicals significantly reduced damage severity and live larval counts relative to the untreated control. At the warmer, lower site, *A. absinthium* and *M. verticillata* achieved large effect sizes, with severity and larval numbers reduced by roughly 80–90% compared with the control, while at the cooler, higher site, larvae were not detected in any botanical treatment. These findings indicate that simple layering of dried leaves from locally available plants, particularly wormwood and muña, can substantially mitigate *S. tangolias* damage in highland seed potato stores and represents a promising, low-cost complement to integrated pest management, although multi-season and dose-response studies are still needed to confirm and refine this approach.

## Simple Summary

Farmers in the Andean highlands face persistent losses of stored seed potatoes due to moth infestations that deteriorate tuber quality and reduce yields. Chemical insecticides are often used to control these pests, but their repeated application increases costs and poses health and environmental risks. In this study, we tested four locally available aromatic plants—*Ambrosia peruviana*, *Eucalyptus globulus*, *Artemisia absinthium* (wormwood) and *Minthostachys verticillata* (muña)—by layering dried leaves inside seed potato bags in two highland villages in Cajamarca, Peru, over a full storage season of 187 days under natural infestation. An entomologist examined the larvae and pupae recovered from damaged tubers and confirmed that all specimens belonged to *S. tangolias*. Compared with untreated bags, all four plants reduced internal damage and the number of live larvae, and wormwood and muña were consistently the most effective, often lowering damage to a small fraction of that observed in the control. These results suggest that a very simple practice—placing modest amounts of dried leaves from locally known plants in seed bags—can help farmers protect their seed potatoes from *S. tangolias* and may reduce the need for synthetic insecticides in highland storage systems.

## 1. Introduction

Potato (*Solanum tuberosum* L.) is one of the most important food crops worldwide, ranking third in human consumption after rice and wheat [1,2,3]. In Latin America, Peru is the leading producer, with an average annual production of 5.4 million tons and a yield of 16.8 t ha^−1^ [4]. Cultivation is widely distributed across 19 of the 25 regions of the country, covering approximately 341,659 ha each year [5]. The economic relevance of potato in Peru is undeniable, as it contributes 6.5% to the Gross Value of Agricultural Production (GVAP) and 9.7% within the agricultural subsector, second only to rice [6].

Within this national context, the region of Cajamarca stands out as the fourth largest potato-producing area, with 28,627 ha under cultivation, following Puno (60,527 ha), Huánuco (44,692 ha), and Cusco (32,250 ha). Potato is cultivated in twelve of Cajamarca’s thirteen provinces, with the highest production concentrated in Cutervo (6681 ha), Chota (5761 ha), Cajamarca (4168 ha), and Celendín (3990 ha) [5]. In this agro-productive context, INIA 302 Amarilis has gained wide adoption due to its resistance to late blight (*Phytophthora infestans*), high yield potential, and excellent cooking quality. Released in 1993 through a joint effort between the International Potato Center (CIP) and the National Institute for Agrarian Innovation (INIA), Amarilis combines productivity with nutritional value, providing significant levels of starch, potassium, and vitamin C [7,8,9].

Despite these advantages, potato production in highland regions is seriously threatened by insect pests, particularly the potato tuber moths *Phthorimaea operculella* [10,11,12] and *Symmetrischema tangolias* [13]. These species are considered among the most damaging storage pests, especially in rustic facilities without refrigeration during warm months [10]. Infestations may reach 100% of stored tubers [14], as females lay more than 100 eggs per life cycle, and the larvae burrow into the tubers, producing galleries [15]. Infested tubers often become infected with secondary pathogens such as fungi and bacteria, leading to rapid deterioration and loss of commercial value [16].

The widespread use of synthetic insecticides has long been the main strategy for controlling *Phthorimaea operculella* and *Symmetrischema tangolias* [15]. However, reliance on chemical inputs increases production costs, pollutes the environment, disrupts populations of natural enemies, and poses risks to farmers and consumers. In addition, smallholder farmers often lack technical guidance for the proper and safe application of pesticides [15]. These limitations highlight the urgent need to develop safer and more sustainable alternatives for pest management in stored potato seed [17].

In response to these challenges, botanical repellents have emerged as promising eco-friendly alternatives to insecticides [18]. Many aromatic and medicinal plants contain bioactive compounds with insecticidal, repellent, ovicidal, antifeedant, or anti-oviposition properties [19,20]. Their use has a long tradition in local farming systems, and in recent decades, research has confirmed their effectiveness in managing insect pests [21]. Approximately 300 plant species have been identified worldwide with proven potential for pest control; for example *Minthostachys* spp., *Eucalyptus globulus*, *Baccharis* spp., *Lantana camara*, *Chenopodium botrys*, *Mentha arvensis*, and *Artemisia vulgaris* have shown activity against *P. operculella* [22]. Similarly, aromatic plants such as *Ocimum basilicum*, *Azadirachta indica*, *Ruta graveolens*, and *Mentha* spp. have demonstrated insecticidal effects against *Spodoptera frugiperda* and other major pests [23]. Therefore, the use of repellent plants has been consolidated as a viable strategy for pest control, with multiple studies highlighting their effectiveness in both field and storage conditions [20].

Here, we conducted parallel storage trials in two highland localities of Cajamarca differing in elevation and associated microclimate to test the effectiveness of four repellent plants applied as dried leaves layered within seed potato bags to control *Symmetrischema tangolias*. We quantified three operational indicators relevant to seed quality management—(i) incidence of moth attack, (ii) damage severity, and (iii) live larval counts—over a six-month storage period. By combining locally accessible botanicals, a widely cultivated variety, and on-farm-like storage conditions across two sites, this study aims to deliver actionable, scalable guidance for smallholder-oriented IPM in Andean potato systems.

## 2. Materials and Methods

### 2.1. Study Sites

**Figure 1:**
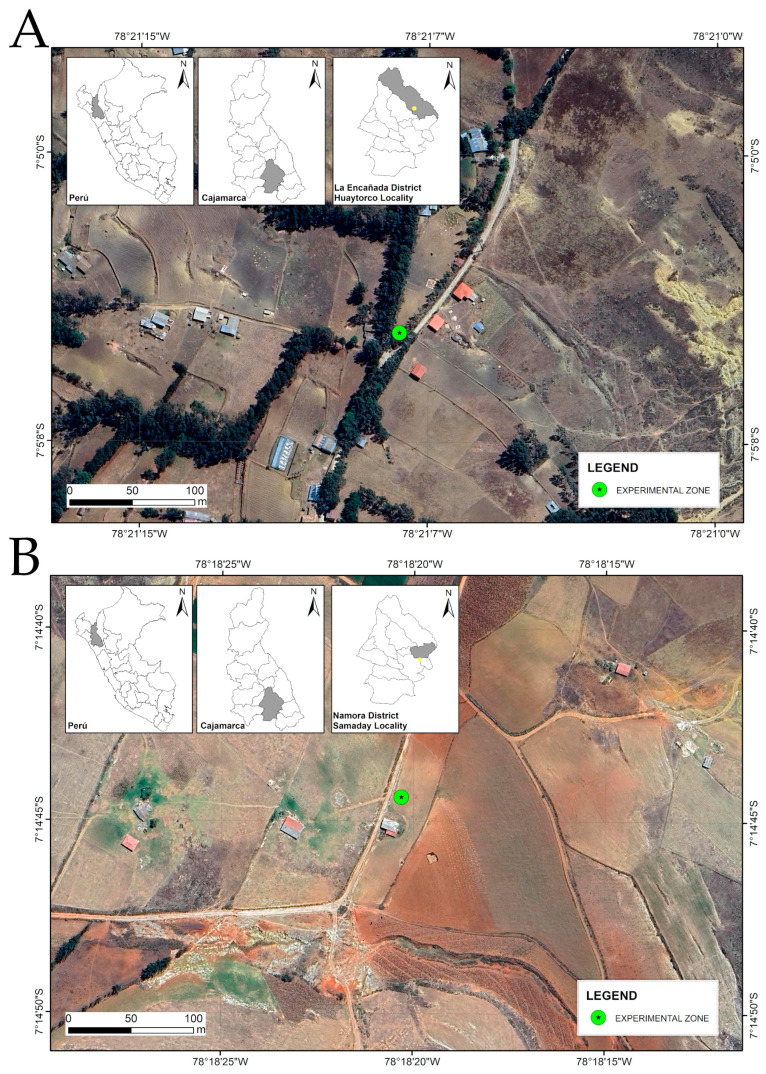
Geographic location of the experimental sites in the localities of (**A**) Huaytorco, La Encañada District, and (**B**) Samaday, Namora District, Cajamarca, Peru. The green dot represents the experimental site, while the yellow dot in the inset maps indicates the province where the experiment was conducted.

The study was conducted during the 2024–2025 storage period in two experimental warehouses located in Cajamarca Province, Peru. The first site was Huaytorco (7°05′04.54″ S, 78°21′08.51″ W; 3350 m a.s.l.), in the district of La Encañada, and the second site was Samaday (7°14′44.37″ S, 78°18′20.56″ W; 2750 m a.s.l.), in the district of Namora (Figure 1A,B).

### 2.2. Climate Data

**Figure 2:**
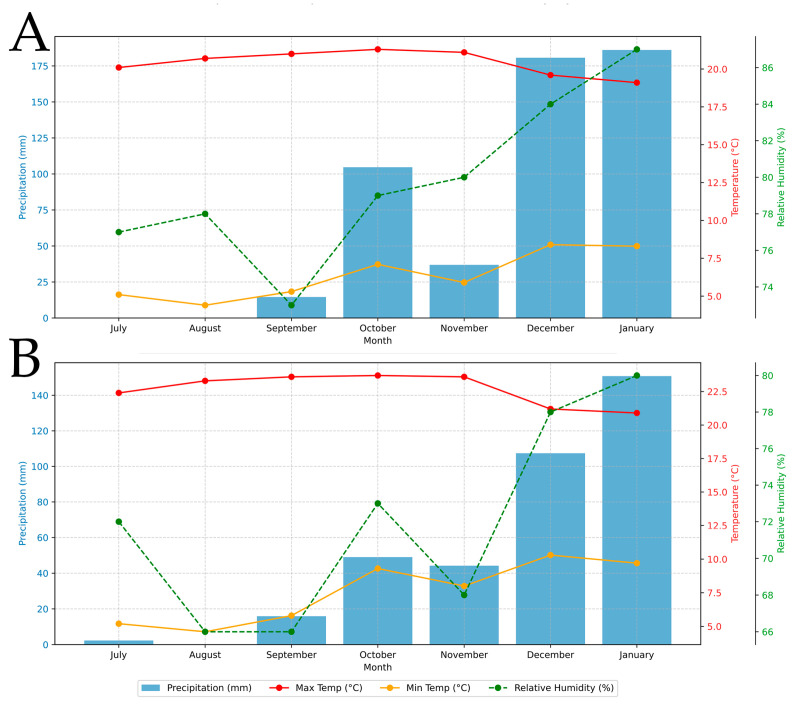
Monthly rainfall, maximum and minimum temperature, and relative humidity recorded during the experimental period (2024–2025) in (**A**) Huaytorco and (**B**) Samaday.

The stores were single-room, naturally ventilated structures without active temperature control; doors/windows remained open during daytime. Meteorological data were obtained from SENAMHI stations located in both study sites during 2024–2025. In Huaytorco, monthly rainfall displayed a progressive increase, from null values in July and August to nearly 186.1 mm in January, with a total accumulated precipitation of 522.9 mm. Maximum temperature remained relatively stable, fluctuating only slightly between 19.1 °C and 21.3 °C (mean: 20.41 °C). Minimum temperature showed an upward trend, rising from 4.4 °C in August to 8.3 °C in January (mean: 6.36 °C). Relative humidity also exhibited a steady increase, rising from approximately 77% in July to 87% in January (Figure 2A). In Samaday, rainfall followed a similar progressive pattern, with a monthly average of 61.21 mm. January stood out as the wettest month (150.8 mm), while August was the driest (0 mm). Maximum temperature was relatively stable, averaging 23.7 °C, with a slight decrease toward the austral summer. Minimum temperature averaged 8.62 °C, with the lowest values recorded in winter (4.6 °C in August) and the highest in December (10.3 °C). Mean relative humidity was 71.85%, gradually increasing toward the warmer months and reaching 80% in January (Figure 2B).

### 2.3. Plant Material

According to Pradel et al. [24], INIA 302 Amarilis is the most widely cultivated potato cultivar in the Peruvian Andean highlands—particularly in Cajamarca, where it accounts for 44% of the total potato area. Nationally, its adoption is estimated at 7.15% [8,9]. Given its broad uptake and agronomic relevance, this cultivar was selected as the reference material for the present study.

In addition to the potato cultivar, repellent plants were incorporated as complementary biological material. These plants were collected directly in both experimental localities, where they grow wild with high availability and easy access for farmers. The identified species included Marco (*Ambrosia peruviana*), Eucalyptus (*Eucalyptus globulus*), Wormwood (*Artemisia absinthium*), and Muña (*Minthostachys mollis*).

### 2.4. Experimental Design

In both localities, a total of 4500 seed tubers, weighing 450 kg in total, were stored and distributed across five treatments (four with the application of dried leaves from repellent plants and one untreated control). The experiment was conducted using a completely randomized design (CRD) with three replicates and five treatments (including the control). The experimental units consisted of 150 tubers per treatment. The trial was carried out under storage conditions characterized by diffuse light, proper ventilation, and a storage area measuring 2 m in width, 5 m in length, and 2.5 m in height. Each experimental unit consisted of a red mesh bag with a capacity of 50 kg, containing 15 kg of seed tubers per bag, resulting in a total of 15 experimental units per locality.

### 2.5. Experimental Management

Repellent plants were collected in the study areas, and the leaves of the selected species were naturally air-dried at room temperature (18–22 °C) in the shade and with good ventilation until reaching an average moisture content of 12%. As a result of dehydration, the fresh biomass weight was reduced as follows: *Eucalyptus globulus* (Eucalyptus) from 10 kg to 5 kg, *Ambrosia peruviana* (Marco) from 10 kg to 6 kg, *Artemisia absinthium* (Wormwood) from 6 kg to 4 kg, and *Minthostachys mollis* (Muña) from 10 kg to 4 kg. The dried material was then packed in mesh bags, properly labeled, and stored until use in the experimental setup.

For each experimental unit, 150 seed tubers were used, with the application of 200 g of dried leaves from the repellent plants. The material was distributed in three layers (base, middle, and surface of the tubers) and stored under diffuse light conditions (Table 1).

### 2.6. Storage Variables Evaluated

Evaluations were conducted at 30 day intervals over 187 days, from 9 July 2024, to 16 January 2025. Data obtained at each interval were systematically recorded, and at the end of the storage period, the accumulated values were analyzed for each evaluated variable.

#### 2.6.1. Incidence of Moth Attack (%)

The number of seed tubers showing visible external damage caused by moth larvae was quantified, characterized by holes in sprouts and surface galleries. The incidence percentage was calculated according to Baca et al. [25].

$$Incidence\;\%=\frac{Number\;of\;damage\;tubers}{Total\;number\;of\;tuber}\times 100$$

#### 2.6.2. Moth Damage Severity (%)

To determine damage severity, affected tubers were cut into four equal sections, each representing 25% of the tuber. The visually estimated percentage of internal damaged area was recorded for each section. The average severity percentage was then calculated as proposed by Baca et al. [25]

$$Severity\;\%=\frac{\sum (\%damaged\;area\;per\;tuber)}{Total\;number\;of\;evaluated\;tubers}\times 100$$

#### 2.6.3. Live Larvae Count

The number of live moth larvae, considering all larval stages, present in the affected tubers was recorded.

### 2.7. Data Analysis

Data were analyzed using R software (version 4.5). Since the assumptions of normality and homoscedasticity were not met, a Kruskal–Wallis test was performed, followed by Dunn’s post hoc test with Šidák correction (α = 0.05).

## 3. Results

### 3.1. Incidence of Moth Attack (%) in Huaytorco and Samaday

**Figure 3:**
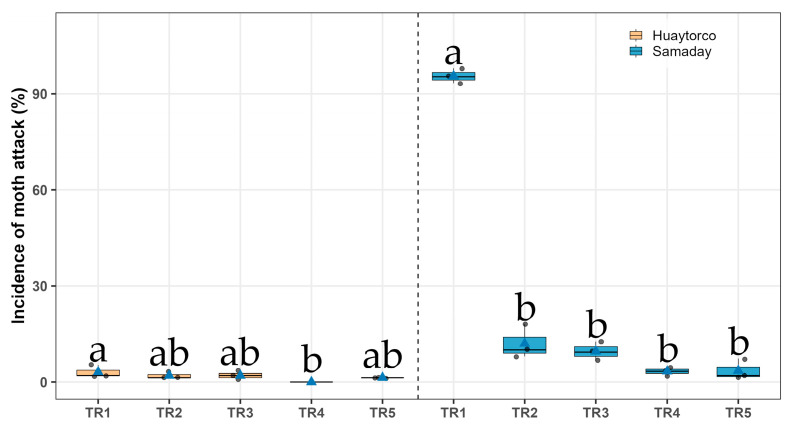
Incidence of moth attack (%) on potato tubers under five treatments (TR1–TR5) across two localities (Huaytorco and Samaday). Data are presented as boxplots; horizontal lines indicate medians, blue triangles represent treatment means, and Gray dots correspond to individual observations. Different letters above the boxes denote significant differences among treatments according to Sidák’s test (*p* < 0.05).

In the locality of Huaytorco, significant differences in damage incidence were observed among treatments (Sidak, *p* < 0.05). However, clear trends were evident. The treatment TR4, consisting of dry leaves of *Artemisia absinthium* (Wormwood) at a dose of 200 g/ 15 kg of seed, recorded the lowest incidence of damage (0.00%). This was followed by TR5 (1.33% ± 0.00), TR2 (1.99% ± 0.67), and TR3 (2.00% ± 0.77). In contrast, control (TR1) registered the highest incidence (3.11% ± 1.11), indicating slightly higher susceptibility to damage compared with the botanical treatments. In Samaday–Namora, significant differences in damage incidence were detected among treatments (Sidak, *p* < 0.05). Once again, TR4 with dry leaves of *A. absinthium* achieved the lowest incidence (3.33% ± 0.77), followed closely by TR5 (3.55% ± 1.89). TR2 (12.00% ± 3.06) and TR3 (9.56% ± 4.06) showed intermediate values. In contrast, the control treatment TR1 exhibited an extremely high incidence (95.55% ± 1.35), representing the most severe level of damage compared with all botanical treatments (Figure 3 and Table 2).

### 3.2. Moth Damage Severity (%) in Huaytorco and Samaday

**Figure 4:**
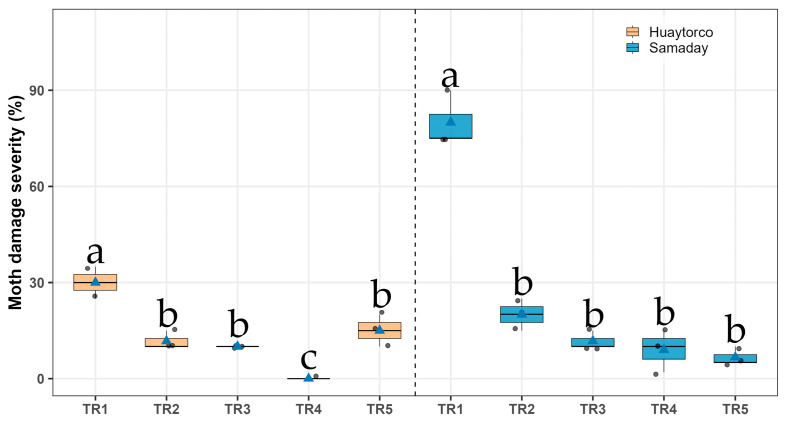
Moth damage severity (%) in seeds potato under five treatments (TR1–TR5) across two localities (Huaytorco and Samaday). Data are presented as boxplots; horizontal lines indicate medians, blue triangles represent treatment means, and gray dots correspond to individual observations. Different letters above the boxes denote significant differences among treatments according to Sidák’s test (*p* < 0.05).

In Huaytorco and Samaday locations, significant differences in damage severity were observed among treatments (Sidak, *p* < 0.05). The treatment TR4 showed no visible severity of damage (0.00% ± 0.00). This was followed by TR3 (10.00% ± 0.00), TR2 (11.67% ± 1.67), and TR5 (15.00% ± 2.89). By contrast, the untreated control (TR1) exhibited the highest severity (30.0% ± 2.89), representing significantly greater damage compared with TR2 (Figure 4). In Samaday, the lowest values were obtained with TR5 (6.67% ± 1.67), TR4 (9.00% ± 3.78), and TR3 (11.67% ± 1.67). In contrast, the control treatment TR1 showed the highest severity level (80.0% ± 5.00), indicating much greater damage compared with TR2 (Figure 4 and Table 2).

### 3.3. Live Larvae Count in Huaytorco and Samaday

**Figure 5:**
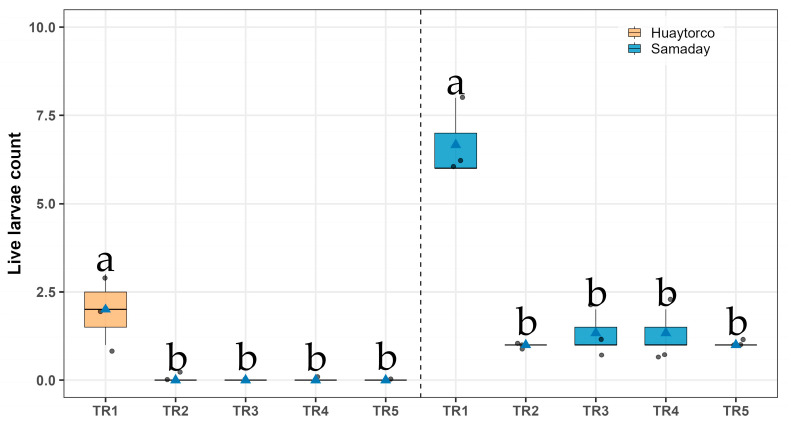
Live larvae count in seeds potato under five treatments (TR1–TR5) across two localities (Huaytorco and Samaday). Data are presented as boxplots; horizontal lines indicate medians, blue triangles represent treatment means, and gray dots correspond to individual observations. Different letters above the boxes denote significant differences among treatments according to Sidák’s test (*p* < 0.05).

In Huaytorco and Samaday, significant differences in the number of live larvae of potato tuber moth (*Symmetrischema tangolias*) were observed among treatments (Sidak, *p* < 0.05). In Huaytorco, treatments TR2, TR3, TR4, and TR5 all recorded no larvae. By contrast, the untreated control (TR1) registered the highest number of larvae (2.00 ± 0.58), indicating a slight presence of pest survival compared with the botanical treatments. In Samaday treatments TR2 (1.00 ± 0.00), TR3 (1.33 ± 0.33), TR4 (1.33 ± 0.33), and TR5 (1.00 ± 0.00) showed very low larval counts. In contrast, the untreated control (TR1) exhibited the highest number of larvae (6.67 ± 0.67), confirming its greater susceptibility compared with the botanical treatments (Figure 5 And Table 2).

## 4. Discussion

Differences in pest pressure observed between the two highland localities can be primarily attributed to altitude-driven microclimatic conditions. In our trials, the higher site (Huaytorco, 3350 m a.s.l.) showed much lower tuber moth damage, whereas the slightly lower site (Samaday, 2750 m a.s.l.) suffered higher incidence, severity and larval counts. This pattern is consistent with the known ecology of the two potato tuber moth species, whereby *Symmetrischema tangolias* tends to thrive in cooler high-altitude environments [26], while *Phthorimaea operculella* usually predominates at lower, warmer elevations in the Andes [27]. In the present trial, however, all morphologically confirmed larvae and pupae corresponded to *S. tangolias*, and *P. operculella* was not detected at either site throughout the 187-day storage period, so the observed elevation-related differences in damage reflect variation in *S. tangolias* pressure rather than species turnover. Monthly climate records for the two districts revealed modest but consistent contrasts in temperature, rainfall and relative humidity, with conditions at the lower site favoring faster insect development and persistence, whereas the cooler and more humid high-elevation environment likely restricted population growth. These findings underscore the importance of considering local climate factors when designing management strategies, as they can significantly influence *S. tangolias* dynamics in farmer-style storage systems [28].

Despite these environmental differences, our field results clearly demonstrate the potential of botanical repellent treatments to suppress tuber moth infestations under high-Andean conditions [29,30]. All tested plant powders provided significant protection compared to untreated controls, with certain species standing out. In particular, *Artemisia absinthium* (wormwood) and *Minthostachys mollis* (Andean mint) consistently achieved the lowest damage levels and almost complete larval suppression. In Huaytorco, these two treatments even prevented any detectable larval survival, an efficacy comparable to or better than conventional chemical control. The performance of *A. absinthium* and *M. mollis* highlights the promise of locally available plants as eco-friendly alternatives to synthetic insecticides [31,32,33]. Notably, *Minthostachys* (known as “muña” in the Andes) and *Artemisia* species have a long tradition of use by farmers for pest repellent purposes, and our data provide quantitative evidence validating their effectiveness in situ [34]. These results are in line with recommendations in the integrated pest management literature that sprigs or powders of aromatic plants like muña, eucalyptus, lantana, and even pepper trees (*Schinus molle*) can be employed to repel potato tuber moths, reinforcing that our tested species are appropriate choices for smallholder use [35,36].

Evidence from previous studies in various regions strongly supports the efficacy of these and other botanical interventions against potato tuber moths. For instance, a classic Peruvian study by Rivera and Retamozo [37] found that *Minthostachys*, *Eucalyptus globulus*, *Baccharis*, *Lantana camara*, *Chenopodium botrys*, *Mentha* (mint), and *Artemisia* could all significantly protect stored tubers from *P. operculella* damage. More recent work in East Africa showed that using dried *Eucalyptus* leaves (50 g per 650 tubers) reduced *P. operculella* infestation to only ~7.2%, a level of protection comparable to the low severity we observed with *E. globulus* treatment [38]. Similarly, dusting potato tubers with onion (*Allium cepa*) bulb powder (50% mixed with talc) was found to drastically reduce moth oviposition and adult emergence, demonstrating that even common kitchen botanicals can confer substantial protection [39]. In our study, the moderate performance of *E. globulus* and *Ambrosia peruviana* (which still reduced damage relative to controls) is consistent with these reports—they offer measurable repellency, though perhaps not as extreme as *A. absinthium* or muña. Nonetheless, the general trend observed across diverse studies is that botanical treatments can markedly lower potato tuber moth infestation levels, often on par with synthetic insecticides, but without the attendant drawbacks.

Our results with *A. absinthium* and *M. mollis* mirror several other experiments under both Andean and non-Andean conditions. Espinoza [40] reported that treating native potatoes in a high Andean storage in Cusco with dried *A. absinthium* kept ~81.7% of tubers completely free of damage, compared to only ~42% protected when using eucalyptus leaves. This aligns closely with our wormwood treatment, which achieved near-total protection, and suggests wormwood’s effectiveness is robust across different highland regions. Likewise, a study in Nepal’s mid-hills by Giri et al. [41] showed that *Artemisia* plant material could cut *P. operculella* tuber infestation roughly in half (36.6% vs. ~70% in untreated stores), and that a local mint relative (*Minthostachys* sp.) reduced damage to around 5% of tubers versus 12% in controls. Notably, in that same study a *Bacillus thuringiensis* (Btk) biopesticide was tested alongside botanicals, and all treatments significantly lowered tuber damage for up to 2–3 months of storage. The *Minthostachys* treatment in particular provided ongoing protection, similar to our findings with *M. mollis*. These cross-regional comparisons (Peru, Ethiopia, Nepal, etc.) underscore a consistent pattern: botanical repellents and biopesticides can yield major reductions in potato tuber moth damage under storage conditions, often approaching the efficacy of chemical controls. Moreover, the effectiveness of our top treatments (*A. absinthium* and muña) even exceeded some literature values—for example, *Minthostachys* powders in another Peruvian trial reduced tuber damage to ~5%, whereas in our case damage was essentially nil in the best scenarios [31]. Such results bolster confidence that these plants hold real potential for protecting stored seed potatoes, especially in rural highlands where conventional pesticide use is limited or undesirable.

Beyond the specific botanicals we tested, a variety of other plant-based materials and organic methods have shown similar promise against tuber moths. Layering or covering stored tubers with dried foliage of certain plants can be highly protective. Lal [42] observed that a 2.5 cm layer of crushed leaves from *Ambrosia*, *Eupatorium*, *Eucalyptus*, or *Lantana* dramatically lowered tuber damage (reducing infestations that exceeded 70% in untreated lots down to about 5%) and that this effect lasted up to six months. Likewise, Chandel et al. [43] in India found that simply adding a layer of wheat straw on stored potatoes created a physical and chemical barrier that kept infestation below 1% over 60 days. Botanical powders made from spices and herbs have also been effective in multiple studies. For example, coriander (*Coriandrum sativum*) and an African shrub (*Zygophyllum*) powder treatments completely prevented larval emergence (0% emergence vs. ~95% in untreated controls) [44]. In an Egyptian trial, powders of clove (*Syzygium aromaticum*), chamomile (*Matricaria chamomilla*), and black pepper (*Piper nigrum*) achieved over 90% larval mortality of *P. operculella*, showing potency comparable to industrial insecticides [45]. Essential oils from oregano (*Origanum vulgare*) have likewise been shown to virtually halt larval development, drastically reducing tuber penetration, pupation, and adult emergence to only a few percent. These diverse approaches–ranging from simple inert covers to aromatic powders and oils–highlight that a wide arsenal of organic and botanical options exist for tuber moth control. In many cases, their performance matches or even exceeds that of conventional insecticides, without leaving toxic residues or driving resistance in pest populations [46]. The common thread is the presence of bioactive compounds or physical deterrents that either repel the adult moths (reducing oviposition) or kill/impair the larvae before they can damage tubers.

It is worth noting that synthetic chemical insecticides, while effective in the short term, have significant limitations in this context. Historically, farmers have relied on broad-spectrum pesticides (e.g., organophosphates or pyrethroids) to protect stored potatoes. These can indeed suppress *P. operculella* infestations initially, but issues such as insecticide resistance, pest resurgence, human health risks, and harm to non-target organisms are well-documented. For example, repeated use of certain chemicals has led to resistant tuber moth populations in some regions, undermining long-term efficacy. Moreover, applying chemicals in rustic storage conditions (often indoors or in bags) poses safety hazards for farmers and can result in undesirable pesticide residues on seed tubers. In recognition of these problems, many countries and research programs now advocate Integrated Pest Management (IPM) approaches that minimize chemical use. In practice, this means combining methods like biopesticides, cultural controls, and botanical repellents. Our study contributes to this shift by providing evidence that botanical powders—a low-cost, locally sourced solution—can effectively replace or reduce the need for synthetic insecticides in highland storage. They are especially attractive for resource-limited farmers because they can be produced on-farm (or collected from the wild), and they pose minimal risk to people or the environment. In an IPM framework, such botanicals could be used alongside other interventions (e.g., pheromone traps, natural enemies, or the granulosis virus biocontrol specific to tuber moths) to achieve sustainable pest suppression. Notably, a baculovirus (“PhopGV”) has been developed and successfully employed in the Andes as a biocontrol agent for potato tuber moths, though its adoption by farmers depends on availability and awareness [47,48]. In comparison, repellent plants offer an immediately accessible tool that farmers are often already familiar with, thus lowering barriers to adoption.

The protective effects observed with *A. absinthium*, *M. mollis* and others can be attributed to the rich cocktail of allelochemicals these plants release. Many aromatic plants have evolved volatile compounds as natural defenses against herbivorous insects. Wormwood (*Artemisia absinthium*), for example, produces an array of monoterpenes and sesquiterpene lactones; its essential oil is particularly rich in camphor and related terpenoids (over 50% of the oil), which are known for their insect-repellent and insecticidal properties [49]. Similarly, *Minthostachys* (muña) leaves contain high concentrations of oxygenated monoterpenes–pulegone (~70–76%) and menthone (~14–20%) are typically the dominant components of *M. mollis* essential oil. Pulegone and menthone are well-documented for their repellent and toxic effects on insects, causing disruption of neuromotor function and deterring feeding or oviposition. When these plant materials are applied to stored tubers (as powders or whole sprigs), they slowly release their volatiles into the storage micro-environment. This can create a protective chemical barrier that confuses or repels adult moths (reducing egg-laying on the tubers) and may also exert contact toxicity or growth inhibition on any larvae that do hatch. The absence of or drastic reduction in live larvae in treated lots suggests that these bioactive compounds interfered with larval establishment and survival. In essence, the plants act as natural fumigants and antipest agents. This mode of action is analogous to certain synthetic products (e.g., mothballs or chemical fumigants), but the key advantage is that botanical volatiles degrade rapidly and leave no harmful residue on seed potatoes. Additionally, because the plants contain multiple active ingredients, the risk of pests developing resistance is lower compared to single-compound synthetic insecticides [50]. Our findings thereby not only confirm the practical efficacy of these repellent plants but also illustrate the underlying biochemical rationale: phytochemicals functioning as repellents, feeding inhibitors, or oviposition deterrents provide a multi-pronged defense.

Looking ahead, future research should prioritize validating these botanical pest control methods across diverse storage conditions and regions, as well as delving deeper into their mechanisms. While our study focused on high-altitude Peruvian settings, similar evaluations in different agro-ecological zones (e.g., lower altitudes, climates, or modern storage facilities) would help determine the broader applicability of repellent plants. It will be important to assess how factors like temperature, storage duration, and pest pressure influence efficacy, and whether combining botanicals with other techniques (such as improved storage structures, biopesticides like *B. thuringiensis*, or pheromone traps) can yield synergistic benefits.

From an applied perspective, the economic feasibility and adoption potential of using local plants should be investigated—for instance, quantifying the cost savings from reduced pesticide use, or evaluating farmers’ willingness to grow/collect these species for pest control. To facilitate wider adoption, the development of standardized formulations (e.g., pre-prepared powders or essential oil sachets) could be valuable, ensuring consistent quality and dosage in the field. A crucial scientific step in that direction is identifying and characterizing the specific bioactive compounds responsible for the repellent and insecticidal effects. By isolating key molecules (such as particular terpenes in muña or absinthin in wormwood) and understanding their modes of action, researchers can optimize mixtures or even breed plant varieties with higher content of these compounds. Such phytochemical studies would also inform safety and regulatory assessments, helping to ensure that botanical treatments are safe for users and do not negatively affect seed potato viability.

Although this study provides operational evidence that layered dry leaves of locally available botanicals can reduce *Symmetrischema tangolias* damage in highland seed potato stores, several limitations should be acknowledged when interpreting the findings and designing follow-up work. First, although inspections were conducted at ≈30-day intervals, our a priori endpoint for statistical comparison was the final cumulative damage at 187 days, so we cannot formally describe temporal dynamics or persistence of efficacy; future trials should analyze the full time series and model early–late storage trajectories. Second, species identification was based on morphological examination by a qualified entomologist (with rearing of ambiguous specimens), and all confirmed individuals corresponded to *S. tangolias* in this single storage season; however, molecular confirmation and multi-season monitoring would provide a more comprehensive picture of the tuber moth complex across years and sites. Third, infestation arose naturally from ambient moth populations under covered, ventilated farmer-style storage, and only two localities were evaluated without within-store blocking, which limits the ability to generalize across agro-ecological zones and to partition the effects of spatial heterogeneity inside stores. Fourth, we relied on nearby weather-station data and qualitative descriptions of storage conditions rather than continuous in-store temperature–humidity logging, precluding robust multivariate attribution of climatic drivers. Finally, we tested a single, practice-based dose (200 g per 15 kg) and did not include a chemical reference treatment, so dose–response relationships and formal benchmarking against registered protectants remain to be quantified. Addressing these limitations through multi-season, multi-site trials that incorporate in-store data loggers, explicit dose–response designs, time-series analyses and a chemical standard, as well as economic and adoption studies, will be essential to confirm, refine and scale the use of botanical leaf layering within integrated pest management strategies for Andean seed potato storage.

Our work and the growing body of literature indicate that locally available repellent plants represent a viable, sustainable tool for managing potato tuber moths in storage. Strengthening the scientific basis and refining the use of these botanical resources will ultimately support their integration into mainstream pest management programs, reducing reliance on synthetic insecticides and enhancing the sustainability of potato production in the Andean highlands and beyond.

## 5. Conclusions

This two-site, 187-day storage study demonstrates that simple layers of dried aromatic leaves provide robust, low-cost protection of seed potatoes against the tuber moth (*Symmetrischema tangolias*) under highland conditions. All four botanicals reduced infestation relative to the untreated control, with *Artemisia absinthium* and *Minthostachys mollis* delivering the most consistent benefits: at the warmer, lower site (Samaday, 2750 m), incidence fell from 95.6% (control) to 3.3–3.6%, severity from 80.0% to 6.7–9.0%, and live larvae from 6.67 to 1.00–1.33; at the cooler, higher site (Huaytorco, 3350 m), *A. absinthium* achieved 0% incidence and 0% severity, while all botanicals suppressed larvae to 0 (vs. 2.00 in the control). *Eucalyptus globulus* and *Ambrosia peruviana* provided intermediate but meaningful protection. Overall, the results provide operational evidence that locally available repellent plant resources can serve as eco-friendly and low-cost alternatives to synthetic pesticides. Their integration into integrated pest management (IPM) programs holds significant promise for enhancing sustainable potato production in the Andean highlands and potentially in other similar agroecological regions.

## Figures and Tables

**Table 1:** Description of the treatments under study.

Treatments	Description	Dose (g/15 kg)
TR-1	Control	---
TR-2	Dried leaves of Marco (*Ambrosia peruviana*)	200 g/15 kg of seed potato
TR-3	Dried leaves of Eucalyptus (*Eucalyptus globulus*)	200 g/15 kg of seed potato
TR-4	Dried leaves of Wormwood (*Artemisia absinthium*)	200 g/15 kg of seed potato
TR-5	Dried leaves of Muña (*Minthostachys mollis*)	200 g/15 kg of seed potato

**Table 2:** Incidence, severity of moth damage, and live larvae count in stored seed potatoes under different repellent plant treatments in two localities. Different letters within each locality indicate significant differences among treatments based on Dunn’s post hoc test with Šidák correction (*p* < 0.05).

Treatments	Location	Incidence (%)	Severity (%)	Live Larvae Count
TR1	Huaytorco	3.11 ^a^ ± 1.11	30.0 ^a^ ± 2.89	2.0 ^a^ ± 0.58
TR2	Huaytorco	1.99 ^ab^ ± 0.67	11.67 ^b^ ± 1.67	0.00 ^b^ ± 0.00
TR3	Huaytorco	2.00 ^ab^ ± 0.77	10.00 ^b^ ± 0.00	0.00 ^b^ ± 0.00
TR4	Huaytorco	0.00 ^b^ ± 0.0	0.00 ^c^ ± 0.00	0.00 ^b^ ± 0.00
TR5	Huaytorco	1.33 ^ab^ ± 0.00	15.00 ^b^ ± 2.89	0.00 ^b^ ± 0.00
TR1	Samaday	95.55 ^a^ ± 1.35	80.0 ^a^ ± 5.00	6.67 ^a^ ± 0.67
TR2	Samaday	12.00 ^b^ ± 3.06	20.00 ^b^ ± 2.89	1.00 ^b^ ± 0.00
TR3	Samaday	9.56 ^b^ ± 4.06	11.67 ^b^ ± 1.67	1.33 ^b^ ± 0.33
TR4	Samaday	3.33 ^b^ ± 0.77	9.00 ^b^ ± 3.78	1.33 ^b^ ± 0.33
TR5	Samaday	3.55 ^b^ ± 1.89	6.67 ^b^ ± 1.67	1.00 ^b^ ± 0.00

## Data Availability

The data are available by contacting corresponding authors for collaboration or other reasonable requests.
